# Malaria Risk Map Using Spatial Multi-Criteria Decision Analysis along Yunnan Border During the Pre-elimination Period

**DOI:** 10.4269/ajtmh.19-0854

**Published:** 2020-06-29

**Authors:** Xiaotao Zhao, Weerapong Thanapongtharm, Siam Lawawirojwong, Chun Wei, Yerong Tang, Yaowu Zhou, Xiaodong Sun, Liwang Cui, Jetsumon Sattabongkot, Jaranit Kaewkungwal

**Affiliations:** 1 Department of Tropical Hygiene, Faculty of Tropical Medicine, Mahidol University, Bangkok, Thailand;; 2 Yunnan Institute of Parasitic Diseases, Pu’er, P. R. China;; 3 Department of Livestock Development, Veterinary Epidemiological Center, Bureau of Disease Control and Veterinary Services, Bangkok, Thailand;; 4 Geo-Informatics and Space Technology Development Agency, Bangkok, Thailand;; 5 Division of Infectious Diseases and Internal Medicine, Department of Internal Medicine, University of South Florida, Tampa, Florida;; 6 Mahidol Vivax Research Unit, Faculty of Tropical Medicine, Mahidol University, Bangkok, Thailand;; 7 Center of Excellence for Biomedical and Public Health Informatics (BIOPHICS), Faculty of Tropical Medicine, Mahidol University, Bangkok, Thailand

## Abstract

In moving toward malaria elimination, finer scale malaria risk maps are required to identify hotspots for implementing surveillance–response activities, allocating resources, and preparing health facilities based on the needs and necessities at each specific area. This study aimed to demonstrate the use of multi-criteria decision analysis (MCDA) in conjunction with geographic information systems (GISs) to create a spatial model and risk maps by integrating satellite remote-sensing and malaria surveillance data from 18 counties of Yunnan Province along the China–Myanmar border. The MCDA composite and annual models and risk maps were created from the consensus among the experts who have been working and know situations in the study areas. The experts identified and provided relative factor weights for nine socioeconomic and disease ecology factors as a weighted linear combination model of the following: ([Forest coverage × 0.041] + [Cropland × 0.086] + [Water body × 0.175] + [Elevation × 0.297] + [Human population density × 0.043] + [Imported case × 0.258] + [Distance to road × 0.030] + [Distance to health facility × 0.033] + [Urbanization × 0.036]). The expert-based model had a good prediction capacity with a high area under curve. The study has demonstrated the novel integrated use of spatial MCDA which combines multiple environmental factors in estimating disease risk by using decision rules derived from existing knowledge or hypothesized understanding of the risk factors via diverse quantitative and qualitative criteria using both data-driven and qualitative indicators from the experts. The model and fine MCDA risk map developed in this study could assist in focusing the elimination efforts in the specifically identified locations with high risks.

## INTRODUCTION

It has been anticipated that Yunnan, particularly the border counties of the province, would be the final location of malaria cases in China at the final phase of malaria elimination. Despite the fact that malaria has been substantially decreasing during the pre-elimination phase (2011–2016), the indigenous cases dropped to zero in 2017, but the imported cases remain a major threat to China’s malaria elimination progress.^1–3^ Spatial and temporal models for predicting malaria and other vector-borne disease risks based on environmental factors such as climate and landscape have been developed for highlighting areas for targeting public health programs.^4–7^ In China, several studies have used spatial and temporal modeling to detect disease clusters while adjusting for population size variation in space and timescales in endemic provinces.^6–14^ Most of the previous spatiotemporal modeling tended to focus on the climatic risk factors such as rainfall, temperature, and humidity related to malaria incidence. At the border regions, using such variables is problematic. First, climatic data are not available on neighboring countries. Second, regional conflicts led to migration and caused malaria outbreaks in the new settlements. An alternative spatiotemporal model of non-climatic, environment-associated risk factors of malaria epidemiology in the border regions may be more appropriate for identifying malaria transmission hotspots and guiding targeted malaria interventions.

There have been several other models and types of input data that can be used to develop a spatial map. For example, a study in Colombia had developed a modeling framework based on geographic information systems (GIS) and remote-sensing environmental data using multiple regression analysis, and subsequently, a model was constructed to estimate the annual parasite incidence and to design risk maps for the entire endemic region.^15^ Another study used binomial logistic regression to examine the determinants of malaria risk among children, and, subsequently, model-based geostatistical methods were applied to analyze, predict, and map malaria prevalence.^16^ Another study used epidemiology and surveillance data to develop and calculate risk factor coefficients via a Bayesian spatial negative binomial model and subsequently used the model-based relative risk estimates to map malaria risk areas.^17^ Multi-criteria decision analysis (MCDA) is a decision support tool that allows for the consideration of diverse quantitative and qualitative criteria using both data-driven and qualitative indicators from stakeholder participations.^4^ Multi-criteria decision analysis was initiated in the field of operation research and subsequently has been applied to many disciplines, including environmental, agricultural, transportation, and urban planning, and recently has been applied in public health.^18–25^ Multi-criteria decision analysis uses statistical methods and human intuition, allows expert interaction, and accommodates nonlinear relationships common between disease organisms and the environment. Multi-criteria decision analysis also allows the combination of multiple environmental factors in estimating disease risk by using decision rules derived from existing knowledge or hypothesized understanding of the factors leading to disease occurrence.^20,26^ Geographic information systems have been widely applied to malaria risk mapping. Geographic information systems in conjunction with MCDA, sometimes referred to as GIS-MCDA or spatial MCDA, could help gain insights on the effects of spatial constraints such as zoning, land use, or demography on program management in public health and other settings.^24–27^ The advantages of the GIS-MCDA model are that it is the evidential reasoning technique providing a structured framework for information exchange among the different stakeholders and reducing the unstructured nature of the problem; and it can be used to support the optimization and rationalization of the decision-making process.^18,19^ The spatial MCDA has been proved to facilitate a systematic and comprehensive way to identify malaria hazard and risk mapping. Studies in Africa and South America applied MCDA and produced risk maps from expert opinions on the spatial risk representation of potential vector exposure and malaria transmission to tackle the challenge during malaria elimination.^5,26–33^

As China is moving toward malaria elimination, the capacity to accurately and reliably map malaria risk and target resources becomes an invaluable resource to ensure program success.^33^ Spatially accurate and fine risk maps could help planning, decision-making, and prioritizing areas for targeted control interventions, but socio-ecological and environmental factors and local malaria transmission patterns might change over the years and could also directly influence the design and delivery of prevention and control strategies. This study thus aimed to identify spatial clusters of malaria and quantify the relationships between risk factors and malaria cases. Specifically, this study focused on the identification of high-risk locations and periods of malaria cases along the border areas of Yunnan Province during its pre-elimination period. This was also the first attempt to integrate satellite remote-sensing and malaria surveillance data to estimate the impacts of socio-environmental factors on malaria cases in persistent endemic areas along the China–Myanmar border.

## METHODS

### Study area.

**Figure 1.**
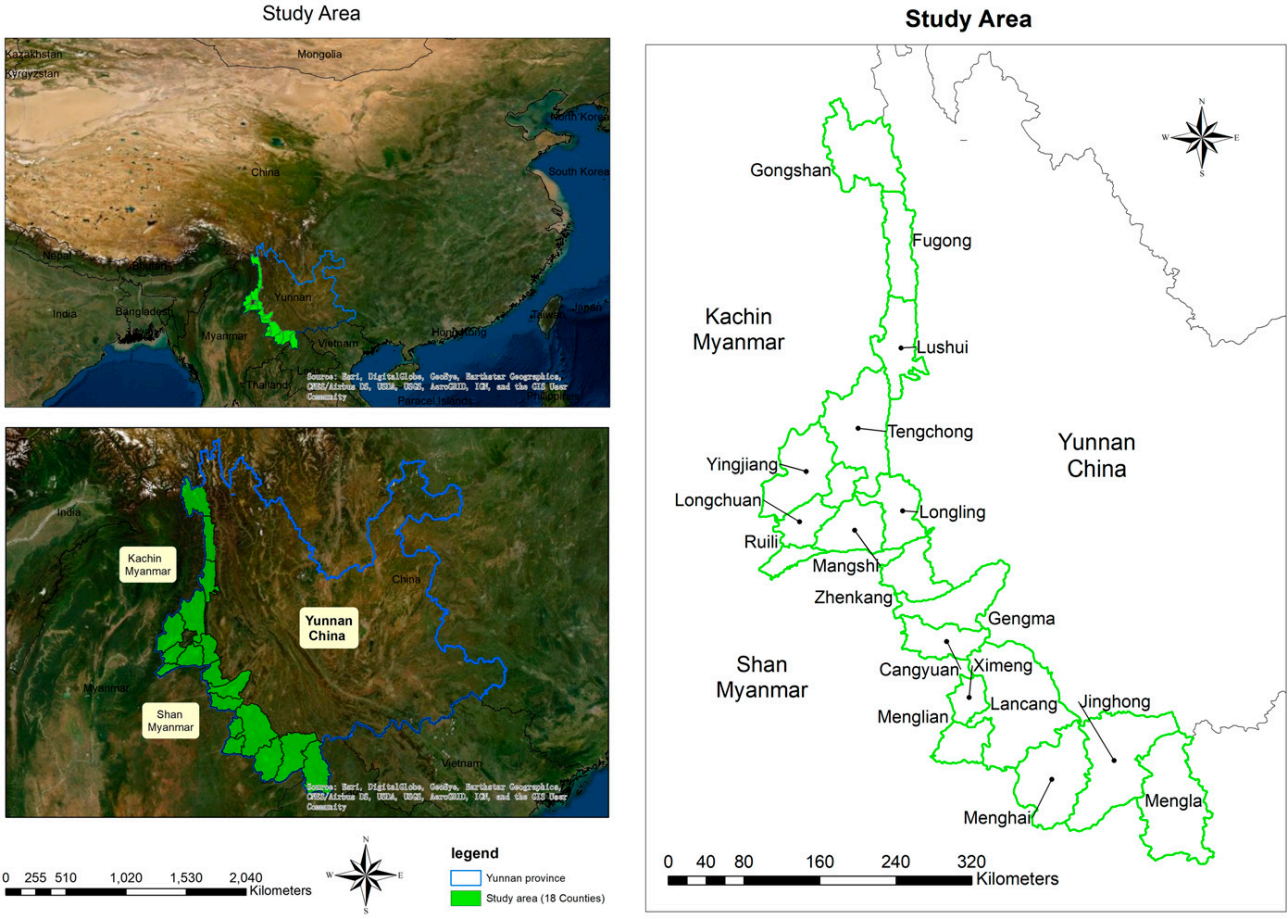
Map of the study area with the 18 counties of Yunnan Province bordering Myanmar highlighted (**A**) and (**B**). This figure appears in color at www.ajtmh.org.

The study area comprises 18 counties along the China–Myanmar border in Yunnan Province (Figure 1). Yunnan is located in southwestern China, bordering Myanmar in the west with a 2,185-km land border. The five Myanmar special regions bordering Yunnan are mountainous and forested. The local residents are mainly ethnic minorities living under poor conditions. This area has an annual average total population of 4.88 million (during 2011–2016). The elevation varies greatly from 210 to 4,878 m above sea level.

### Multi-criteria decision analysis.

The spatial MCDA model used here encompassed five steps: 1) identify the risk factors, 2) identify relative importance of each risk factor, 3) standardize the factors for comparability, 4) perform multi-criteria evaluation (MCE), and 5) validate the model (accuracy assessment).

#### Risk factor identification.

Malaria risk factors commonly include hosts, vectors, pathogens, and environment, which vary in space and time. Based on literature review, besides climatic factors (temperature, rainfall, humidity, etc.), non-climatic factors influencing malaria distribution and transmission include, for example, environmental changes, socioeconomic status, human activities, and population movement and settlement (density).^34–39^ This study focuses on the non-climatic risk factors of malaria transmission at the local level in the Yunnan border areas.

In developing the MCDA model, risk factors can be identified and achieved either by extracting information from a literature review or from expert opinions.^40,41^ In this study, as there has been no similar research in this area and ranking for relative importance and type associated with risk factors (standardization) could not be achieved precisely through literature review, an expert opinion method was selected. The working process of expert opinion included 1) making a list of risk factors of malaria and 2) brainstorming and obtaining consensus on the weightings of each risk factor.^41^

There were six experts in Yunnan Institute of Parasitic Diseases who passed the inclusion criteria such that they had experiences in malaria control and specialized in fieldwork, laboratory work, or project management (Table 1). They were invited to a brainstorm session to express their opinions and reach a consensus on a pair-wise comparison matrix by using the analytical hierarchy process (AHP).

**Table 1:** Information about the experts in Yunnan Institute of Parasitic Diseases

Number	Professional title	Years working on malaria	Expertise
1	Senior	33	Laboratory
2	Senior	30	Epidemiology and vector control
3	Senior	20	Epidemiology
4	Intermediate	13	Epidemiology
5	Intermediate	10	Vector control
6	Intermediate	9	Project management

Based on the discussion of potential risk factors in the study areas, the six experts had a consensus on nine risk factors, as shown in Table 2. Data sources of the risk factors on both China and Myanmar sides were extracted from remote-sensing online resources in certain official websites. GPS data of villages and health facilities in China were obtained from official websites. The primary data used in this study, listed in Table 2, were extracted from different data sources. Land cover data, including forest coverage, cropland, and water body, were extracted from the GLOBELAND30 website (http://www.globallandcover.com/GLC30Download/index.aspx), which provides global raster maps of land cover with 30-m resolution. The SRTM digital elevation data with 90-m resolution, produced by NASA, were extracted from the CGIARCSI website (http://www.cgiar-csi.org/data). The human population density raster dataset at 100-m resolution was obtained from the WorldPop project (http://www.worldpop.org.uk/). The road vector data were downloaded from the DIVA-GIS website (http://www.diva-gis.org/), and the health facility coordinates were downloaded from the GPSspg website (http://www.gpsspg.com/). The distance to road and distance to health facility data were processed in ArcGIS 10.2 (Redlands, CA) using the cost distance tool. Urbanization data were extracted from the SEDAC website (http://sedac.ciesin.columbia.edu/data/set/grump-v1-urban-ext-polygons-rev01/data-download). Data on malaria cases and demographic data were collected from 613 villages (all villages with reported malaria cases during the study period) in 18 counties of Yunnan Province from 2011 to 2016. Population data at the village level were obtained from the local governments on the China side. Imported cases of malaria, collected by the National Notifiable Infectious Disease Reporting Information System (NIDRIS) of China, were aggregated into polygons of 18 counties bordering Myanmar. All geographical data were converted into raster datasets with 100-m resolution; ArcGIS 10.2 software was used to manage all geo-processing.

**Table 2:** Risk factors for malaria according to expert consensus

Risk factor	Description
Forest coverage	Forest coverage in 18 counties of Yunnan Province bordering with Myanmar. Data extracted from: http://www.globallandcover.com/GLC30Download/index.asp
Cropland	Cultivated land coverage in 18 counties of Yunnan Province bordering with Myanmar. Data extracted from: http://www.globallandcover.com/GLC30Download/index.asp
Water body	Water bodies in 18 counties of Yunnan Province bordering with Myanmar. Data extracted from: http://www.globallandcover.com/GLC30Download/index.asp
Elevation	Elevation in 18 counties of Yunnan Province bordering with Myanmar. Data extracted from: http://www.cgiar-csi.org/data/srtm-90m-digital-elevation-database-v4-1
Human population density	Population in 18 counties of Yunnan Province bordering with Myanmar. Data extracted from: http://www.worldpop.org.uk/
Imported case	Monthly imported cases at the village level in 18 counties bordering with Myanmar during 2011–2016. Data extracted from the National Notifiable Infectious Disease Reporting Information System of China
Distance to road	Main road development in 18 counties of Yunnan Province bordering with Myanmar. Data extracted from: http://www.diva-gis.org/
Distance to health facility	GPS data (point) of township-level health facilities (the lowest level health facility where the malaria can be diagnosed, treated, and reported) in 18 counties. Data extracted from: http://www.gpsspg.com/
Urbanization	Land use in 18 counties. Data extracted from: http://sedac.ciesin.columbia.edu/data/set/grump-v1-urban-ext-polygons-rev01/data-download

#### Relative importance weight of each risk factor.

In assessing the importance of the selected factors, the AHP was used. In the AHP, the experts will assign and have a consensus on weight of each factor by assessing its importance relative to the importance of the other factor in pair using a pair-wise matrix.^42^ As shown in Table 3, the importance of each factor relative to the other in a pair would have a value ranging from 1 (extremely less important) to 9 (extremely more important). The consistency of the pair-wise matrix is then evaluated as follows: consistency ratio (CR) = CI/RI, where CI = (λ_max_−*n*)/(*n*−1), RI is the random consistency index (shown in Table 4), *n* is the number of factors, CI is the consistency index, and λ_max_ is priority vector multiplied by each column total. The CR of the pair-wise matrix was evaluated using CR = 0.1 as a threshold. If CR > 0.1, then some pair-wise values required revision until CR < 0.10 was reached, indicating an acceptable consistency.^42,43^

**Table 3:** A nine-point continuous comparison scale

Less important					More important			
Extremely	Very strongly	Strongly	Moderately	Equally	Moderately	Strongly	Very strongly	Extremely
1/9	1/7	1/5	1/3	1	3	5	7	9

**Table 4:** Random indices for matrices

Number of factors	1	2	3	4	5	6	7	8	9	10	11	12	13	14	15
RI	0.00	0.00	0.58	0.90	1.12	1.24	1.32	1.41	1.46	1.49	1.51	1.54	1.56	1.57	1.58

#### Factor standardization.

The data layers might contain variably scaled information; thus, fuzzy functions are used to standardize all the layers to a common data range needed to facilitate factor integration. Fuzzy functions measure the degree of membership of data cells in a layer through control points that are set based on the relationship between the layer and disease/vectors. In the ArcGIS program, these relationships determine the shape (linear, Guassian, large, small, etc.) and direction (increasing, decreasing, or symmetric) of the fuzzy function. In this study, those functions were selected and represented on an 8-bit (0–255) scale in the data analysis.

#### Multi-criteria evaluation.

Subsequently, the MCE procedure is used to integrate all data layers to create composite risk maps for the study area by choosing the weighted linear combination (WLC). The WLC is a linear function which combines fuzzy layers according to their weight of importance (i.e., all factor weights add up to 1).^41,44^

$$S=\sum_{i=1}^{n}w_{i}x_{i}c_{j},$$

where $w_{i}$ = the weight of factor i, $x_{i}$ = the criterion score of factor i (value corresponding to the raster cell in the criterion raster map), *n* = the number of factor, and $c_{i}$ = the criterion score (1 or 0) of constraint.

#### Map validation (accuracy assessment).

**Figure 2.**
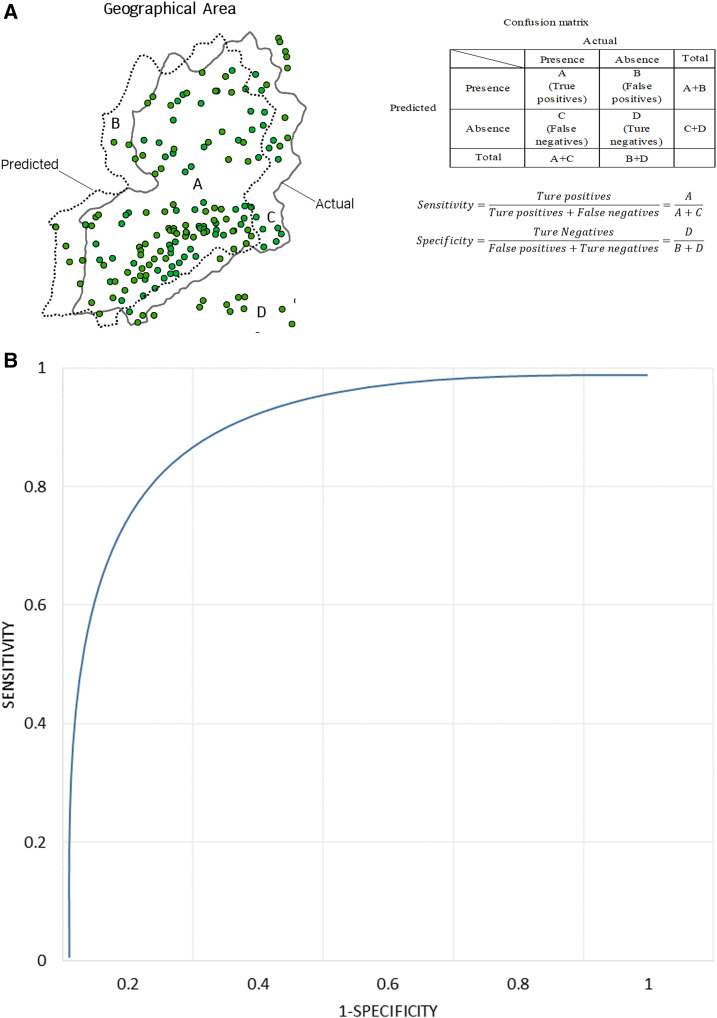
Model validation of multi-criteria decision analysis. (**A**) Formula of sensitivity and specificity and (**B**) area under the curve of receiver operating characteristic plot. Note: Sensitivity and specificity: 0.5–0.7 indicates low accuracy, 0.7–0.9 indicates useful applications, and > 0.9 indicates high accuracy. This figure appears in color at www.ajtmh.org.

The predictive ability of the MCDA models can be quantitatively evaluated by calculating the area under the curve (AUC) from receiver operating characteristic (ROC) analysis (Figure 2). Predicted values are obtained from the suitability maps by calculating the average risk in the study area, whereas observed values correspond to malaria data at the same aggregation level (village). In Yunnan, malaria data during 2011–2016 were obtained from NIDRIS, which has been considered as a surveillance system with very good sensitivity and specificity. Therefore, the model validation was performed by comparing the model performance of experts’ opinion as sources of a priori knowledge for spatial MCDA against the Yunnan dataset to determine the accuracy of the model developed. The AUC of model reflects the suitability of the risk maps for malaria transmission in the study area. In a quantitative validation of the risk map produced in study area, if the predictive performance of the model is useful for prediction, then it should have the AUC with a relatively high predictive power. As a rule of thumb, an AUC of 0.5–0.7 indicates low accuracy, 0.7–0.9 useful applications, and > 0.9 very high accuracy.^45,46^ For example, if the AUC of the model = 0.88, then it means for 88% of the time, a randomly selected location with malaria has a probability that is greater than that for a randomly selected location without malaria.

**Figure 3.**
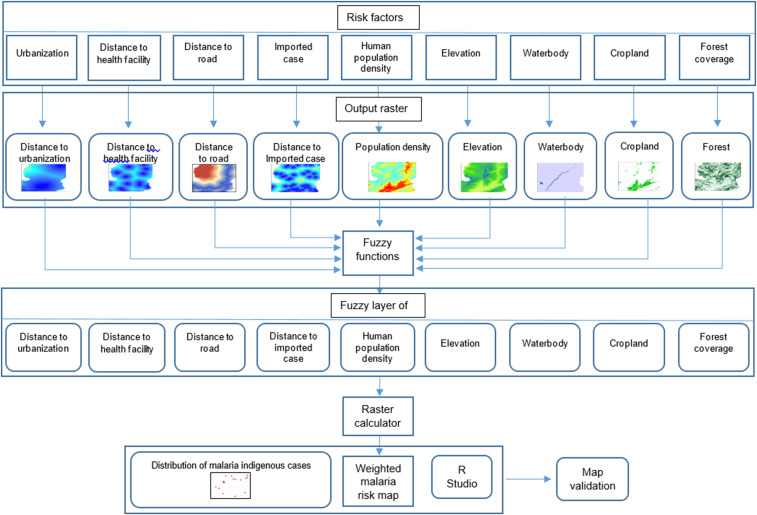
Summary of malaria risk analysis process in ArcGIS and RStudio. This figure appears in color at www.ajtmh.org.

In summary, in developing spatial MCDA models, the five steps were conducted by using the raster-based ArcGIS software and validated in RStudio (Figure 3). As the number of imported cases was the only risk factor that varied over the 6 years of the study period, the analysis at each year was performed to determine any temporal trend changes.

## RESULTS

### Identification and factor weights of malaria risks.

Based on the brainstorm session experts’ consensus on a pair-wise comparison matrix using the AHP, nine malaria risk factors associated with malaria transmission were considered. Table 5 shows a 9 × 9 comparison matrix of the malaria risk factors with factor weights. A value of 1 means the pair of column and row factors has the same weight and affects the malaria occurrence equally. A value of 3, 5, 7, or 9 means the factor in the column has the selected corresponding multiplication weight of important level as a risk of malaria occurrence relative to its pair-wise factor in the row. The weights of each factor used for the spatial model in producing the malaria risk map were as follows: elevation (29.7%), imported cases (25.8%), distance to a water body (17.5%), cultivated land (8.6%), human population density (4.3%), forest coverage (4.1%), urbanization (3.6%), distance to a health facility (3.3%), and distance to road (3.0%). The CR for the pair-wise matrix passed the acceptable threshold with a score of 8.5%, meaning that the factor weighting produced an acceptable consistency.

**Table 5:** Risk factors weighted by experts

	Risk factor	Forest coverage	Cropland	Water body	Elevation	Human population density	Distance to imported case	Distance to road	Distance to health facility	Urbanization	Weight
1	Forest coverage	1	1/3	1/3	1/9	3	1/9	1	1	1	0.041
2	Cropland	3	1	1	1/3	3	1/3	3	1	1	0.086
3	Water body	3	1	1	1/3	3	1	9	9	9	0.175
4	Elevation	9	3	3	1	9	1	9	9	9	0.297
5	Human population density	1/3	1/3	1/3	1/9	1	1/7	1	5	1	0.043
6	Imported case	9	3	1	1	7	1	9	9	9	0.258
7	Distance to road	1	1/3	1/9	1/9	1	1/9	1	1	1	0.030
8	Distance to health facility	1	1	1/9	1/9	1/5	1/9	1	1	1	0.033
9	Urbanization	1	1	1/9	1/9	1	1/9	1	1	1	0.036
	Sum	28.333	11.000	7.000	3.222	28.200	3.921	35.000	37.000	33.000	1.000

### Standardization of malaria risks and mapping by each risk.

**Figure 4.**
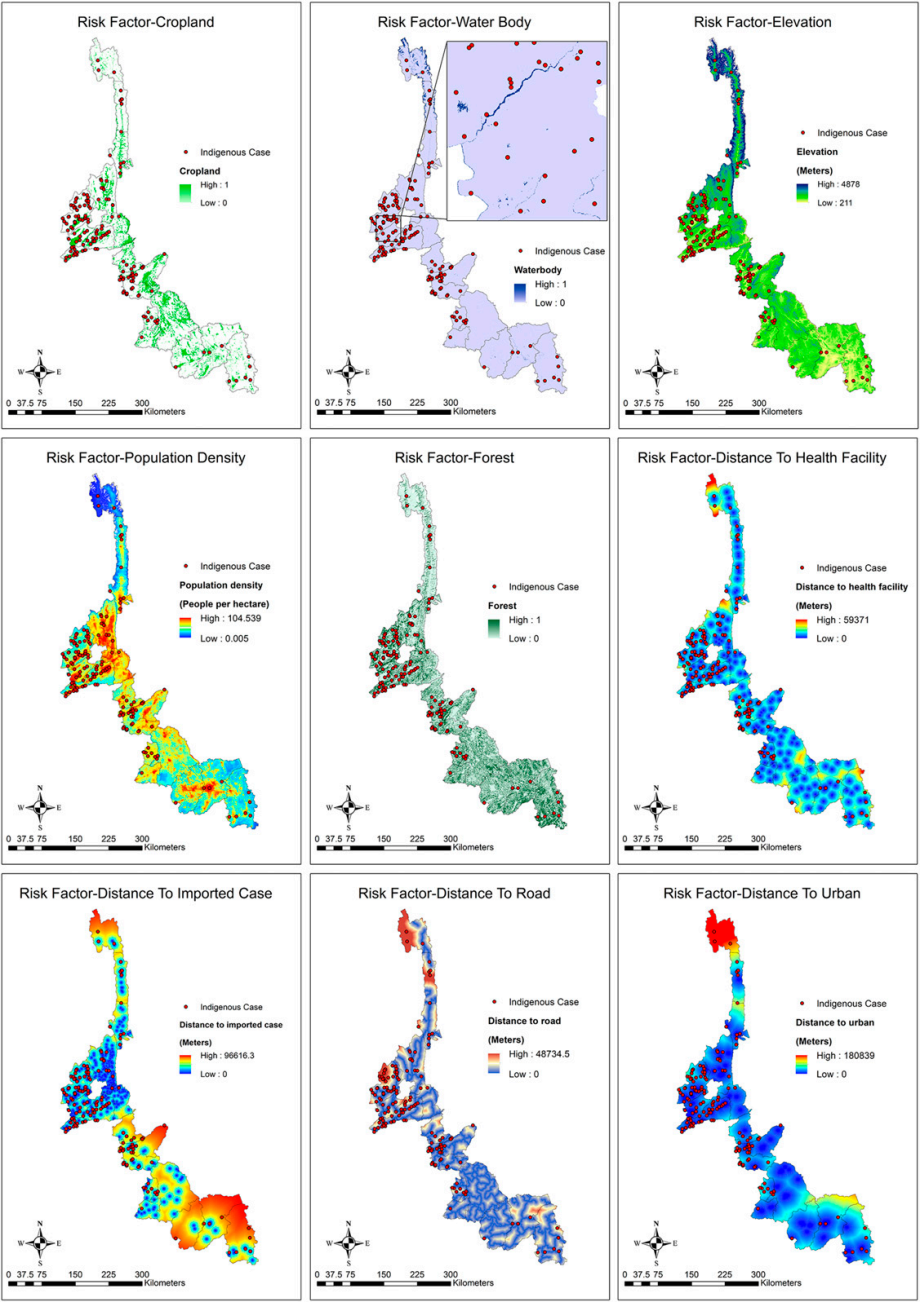
Spatial risk assessment of malaria occurrence by each factor in 18 counties of Yunnan Province. This figure appears in color at www.ajtmh.org.

The data for malaria risk factors were standardized for mapping in the study area using the malaria risk layer as indicated in Table 6. In this study, the fuzzy function for ArcGIS mapping used the WLC approach. Spatial risk assessment and mapping of malaria occurrences in the 18 counties of Yunnan Province by each of the nine factors are shown in Figure 4. In each risk factor map, the locations of indigenous cases were also plotted to visually observe the distribution of cases, with the individual risk factor as a background image.

**Table 6:** Standardization of the selected risk factors

Risk factor	Factor weight	Application in this study	Relationship and control point	Fuzzy function applied in ArcGIS	Assumption/logic
Forest coverage	0.041	Use forest coverage	Binominal ↓, 0, 1 (without, with)	Linear ↓	Forest is not suitable for vector breeding. (Vectors occur in lowland, foothills, and mid-hills some distance away from forest.)
Cropland	0.086	Use cropland coverage	Binominal ↑, 0, 1 (without, with)	Linear ↑	Cropland (paddy field) is suitable for vector breeding
Water body	0.175	Use distance to water body	Binominal ↑, 0, 1 (without, with)	Linear ↑	Vectors found within 2 km of water bodies (normal flight range)
Elevation	0.297	Use elevation	Sigmoidal ↓, 600, 3,500 m (min, max)	Linear ↓	Main vector exposure above 600 m, then decreases with elevation increases, and is null above 3,500 m in the study area
Human population density	0.043	Use predicted human population in 2020	Sigmoidal ↑, 0.005,104.539 unit (min, max)	Linear ↑	Increased population density and greater malaria risk
Imported case	0.258	Use distance to imported cases	Sigmoidal ↓, 0, 5,000 m (min, max)	Linear ↓	Higher risk of malaria transmission within 5 km of imported case (normal flight range of mosquito and human movement)
Distance to road	0.030	Use distance to road	Linear ↑, 5,000, 48,734.5 m (min, max)	Linear ↑	Low risk of malaria infection as higher probability of prevention and control when distance from road is < 5 km
Distance to health facility	0.033	Use distance of health facility	Linear ↑ 10,000, 59,371 m (min, max)	Linear ↑	Low risk of malaria transmission within 10 km of health facility, as individual patient could access timely diagnosis and treatment
Urbanization	0.036	Use distance to urban areas	Sigmoidal ↑ 5,000, 180,839 m (min, max)	Linear ↑	Vectors absent from urban areas but increased in urban periphery and rural areas

### Composite malaria risk mapping by MCE.

**Figure 5.**
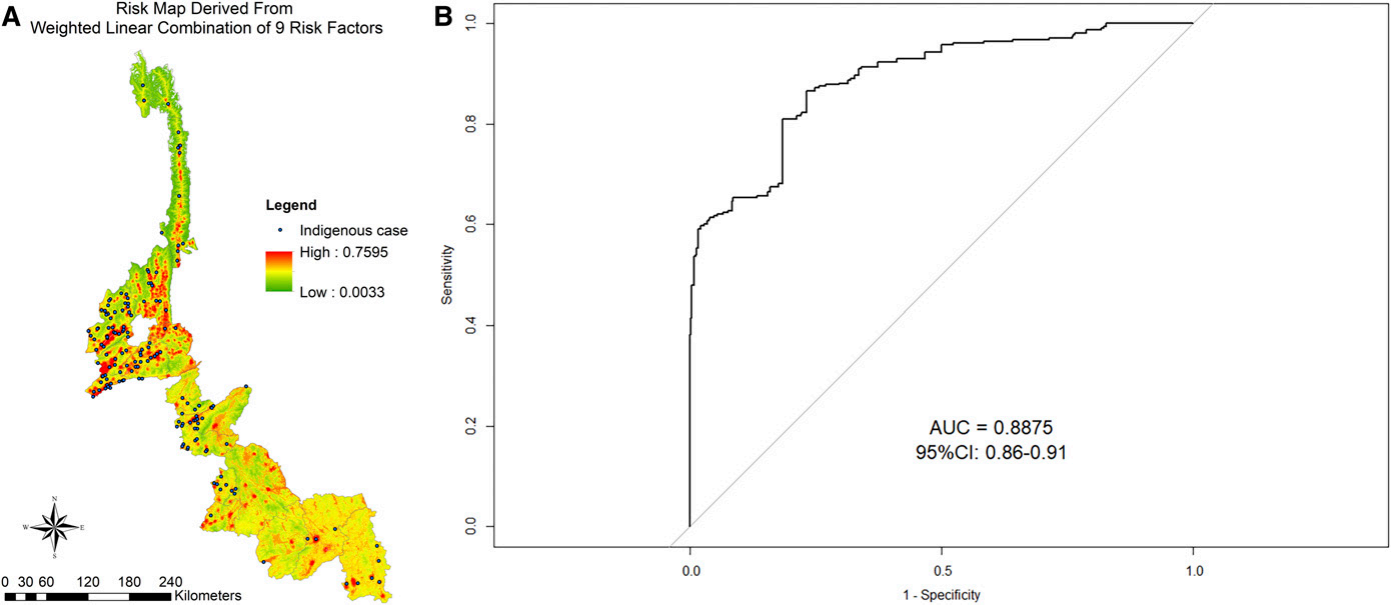
Composite risk map derived from weighted linear combination of nine risk factors. (**A**) Risk areas in 18 counties of Yunnan Province and (**B**) area under the curve of the developed model. This figure appears in color at www.ajtmh.org.

In creating the composite risk map in the study area, the factor weights of malaria risk layers indicated in Table 6 were combined by the WLC method. The model to produce the malaria risk map formula was as follows: [(Forest coverage × 0.041) + (Cropland × 0.086) + (Water body × 0.175) + (Elevation × 0.297) + (Human population density × 0.043) + (Imported case × 0.258) + (Distance to road × 0.030) + (Distance to health facility × 0.033) + (Urbanization × 0.036)]. Figure 5 shows the final malaria risk map of the study area after combining all the weighted risk factor layers by using the Raster Calculator in an ArcGIS environment. The composite risk maps of the nine factors are shown in Figure 5A. In validating the risk map developed comparing the model based on the experts’ opinion against the malaria data in Yunnan’s dataset, the expert-based model for this study had a good prediction capacity with an AUC of 0.89 (CI %95: 0.86–0.91; Figure 5B).

Based on the risk map developed, risk areas can be pinpointed. It can be seen that in the combined map, the high malaria risk areas are mainly concentrated at Yingjiang, Tengchong, Longchuan, Ruili, and Mangshi counties in the western part, and Gengma, Cangyuan, Ximeng, and Menglian counties in the southwestern part of Yunnan Province, which border with KSR II and Shan State of Myanmar, respectively. All these areas are located within low to moderate elevation, close to the major water bodies, and/or close to villages (points) where indigenous cases occurred (Figure 5A).

### Spatial malaria risk mapping by MCE.

**Figure 6.**
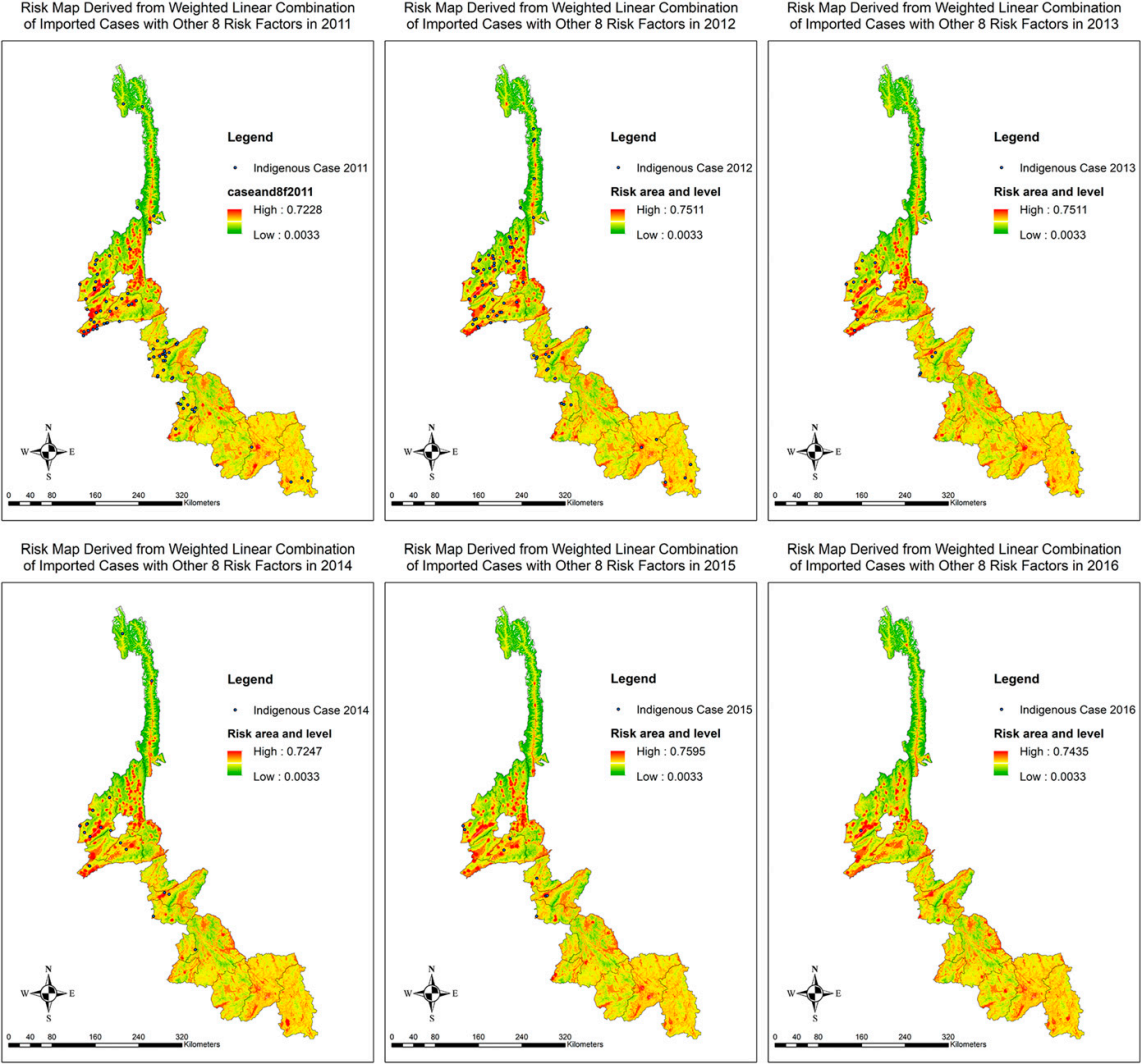
Risk map derived from weighted linear combination of annually imported cases with other eight risk factors. This figure appears in color at www.ajtmh.org.

As a major risk factor was the “imported cases” and it was the variable that changed in terms of the numbers and locations annually over the 6-year study period, it was of interest to develop risk maps by taking into consideration the imported cases as a time-dependent factor. Annual risk maps were created using the same MCDA model but applying the imported cases in each year in combination with the other eight risk factors (Figure 6). The risk maps were validated to check the strength of association between locations where indigenous cases really occurred (from the surveillance system) and the risk areas that was generated by the MCDA model with imported cases that were changed annually in a spatial pattern (Table 7). The AUCs for the risk maps showed good fit of the models, ranging from 0.75 to 1.00. The AUC for the year 2016 was 1.00 because there is only one indigenous case left in the study area. Theoretically, perfect prediction is a point when the predicted and actual values coincide 100%. When examining the risk areas over the 6 years, there appears to be a spatially distributed relationship between the location of the annual indigenous cases and the imported cases. Although both indigenous and imported cases were decreasing, similar risk areas remained in the counties as shown in the composite risk map. No obvious temporal effect was observed as the annual risk areas appeared not to change much over the years.

**Table 7:** Area under the curve of risk map using annually imported cases combined with eight other risk factors in the study area

Year	Indigenous case	Imported case (from Myanmar)	AUC	95% CI
2011	145	432	0.82	0.78–0.85
2012	92	274	0.83	0.79–0.88
2013	40	232	0.75	0.67–0.83
2014	27	235	0.86	0.77–0.96
2015	9	305	0.84	0.67–1.00
2016	1	183	1	1.00–1.00

AUC = area under the curve.

## DISCUSSION

China, particularly Yunnan Province, is now moving toward the malaria elimination phase. As malaria transmission declines, the number and size of the infection foci are shrinking. Strategies to achieve and maintain malaria elimination should concentrate on identifying and eliminating transmission foci. Thus, finer scale malaria risk maps are required to identify transmission hotspots that can be used for implementing surveillance–response strategies, allocating resources, and preparing health facilities based on the requirement of each area. This study used the MCDA model, aiming to identify and assess the relationships between non-climatic factors and malaria cases and to visualize spatial high-risk areas of malaria transmission on a finer scale.

In this study, the MCDA model and risk maps were created from the consensus among the experts who have been working and know malaria situations in the study areas very well. This study not only developed a composite spatial MCDA model but also explored temporal effects by developing annual MCDA risk maps by varying the numbers and locations of annual imported cases in the study areas. The composite model and annual models were validated and appeared to provide a good fit of the model with high AUCs. This good predictive performance could be explained that although the major risk factor, imported cases, changed in terms of the numbers and locations, its weight remained unchanged, and thus the risk area generated by the model for each year was highly consistent with the actual location of indigenous cases that was extracted from the surveillance system. As aforementioned, the possible explanation for the very good performance of the expert-based model was that the experts could identify accurate and precise risk factors used in generating the risk map because they have been working in the study areas for a long time and knew situations in their areas, and some of them had previously conducted research studies in malaria prevention and control. Although imported cases were considered to give the temporal dimension of the study, other variables in the model, which were identified by the experts, represented only the spatial dimension because of the limited availability of data on the websites and limited access to various data sources in China. Our model is thus characterized as a spatial MCDA model rather than a full-fledged spatiotemporal MCDA model. Even though our model provides a good fit for the results, as shown in AUC statistics, the model may be more complete and provide even better fit if these variables and other variables with temporal variation of malaria risk are taken into account.

The selection of malaria risk factors is essential for the reliability of the risk map to be developed. Malaria could be considered as an environmental disease, as the vectors and parasite populations are influenced by specific habitats, humidity, and temperature, whereas the hosts are found to be related to poverty, migration, and access to health care.^5,26,29,47–49^ The experts in this study reached consensus on selecting nine major risk factors for the study areas—forest coverage, cropland, water body, elevation, urbanization, human population density, distance to road, distance to health facility, and imported case. Several other studies using an MCDA model in other settings used similar and different determinants. A study in Mozambique identified malaria risk factors of average temperature, precipitation, altitude, slope, distance to water body, distance to road, normalized difference index, land use and land cover, malaria prevalence, and population density.^32^ A study in Ethiopia used natural factors affecting mosquito-breeding sites, habitat formation, and disease transmission, including distance from water bodies, temperature, drainage status, elevation, and slope; the study also developed malaria risk hazard mapping at a specific hydropower development site using the natural conditions and the other elements at risk, including gross population density, land use/cover, health services, and road access as factors of development.^27^ Spatial MCDA study in Ghana produced a predictive model from eight risk factors ranging from environmental to anthropogenic (human-induced) variables such as land use/land cover changes and distance to road,^29^ whereas another study in Iran used four factors including temperature, water bodies, humidity, and vegetation suggested spatiotemporal variations in trend of malaria-risk area.^47^ Similar to the present study, a study in South America used nine parameters associated with the environment related to availability of vector-breeding sites (wetlands, precipitation, and topographic wetness index), thermal and altitudinal limits for parasites and vectors (elevation and temperature), and access to blood meals (population density, roads, urban areas, and deforestation).^26^ In this study, the experts did not identify climate as a risk factor of malaria incidence and transmission corresponding to the literature review that climatic indicators were rather controversial; some studies reported significant associations, whereas others did not.^30,31,47,48^

The factors identified by the experts in this study reflect the complexity of border malaria transmission, which integrate influential natural factors (i.e., elevation and water body), anthropogenic elements (i.e., forest coverage, cropland, and urbanization), access to healthcare dimensions (i.e., availability and distance to health care), and imported infections (i.e., cases imported along the borders). The integrated risk parameters of natural factors and anthropogenic elements could be seen as land cover and land use characteristics. Land cover concerns the physical material observed at the earth’s surface, whereas land use is related to the human use of the land, integrating socioeconomic and cultural functions.^50^ A focus on land cover helps understand the presence of vectors and hosts, and a focus on land use identifies which places people visit for specific activities, at what time of the day and the year, and how frequently.^27,28,51^ Several studies have noted that land cover and land use were the major eco-epidemiological systems that could improve the understanding and prediction of disease risk.^50,52,53^ Spatial and temporal variations in malaria risk depend not only on land cover but also on land use, via the probability of contact between vectors and hosts.^51^ It should be noted, however, that the elements of land cover and land use might interact with each other, for example, the intensification of deforestation that is generally associated with urbanization or large cultivated areas. Studies with geographical analysis noted that land surfaces covered in low vegetation were associated with a significant malaria risk immediately after deforestation, but lower risk was found when the size of the areas was increasing or when the areas became urbanized.^50,54,55^ Besides the land cover/use factors, the collegial factors, including imported infections and access to health facilities, were also suggested in the literature to be important elements for deploying and targeting efforts and resources, especially while moving toward malaria elimination.^56–62^

The challenge along the border areas is related to the natural environment, which is complex and harbors a variety of malaria vectors.^63^ The land cover natural risk factors identified by the experts were elevation and water body. Topography (elevation) generally has a great influence on mosquito replication such that higher topographies in cooler temperatures would limit the reproduction rate of the parasite.^28^ Studies on environmental factors and malaria risk reported that different mosquito species with special ecological niches, and slope, play important roles in the distribution of malaria vectors.^49,64,65^ Previous studies reported the relationship of elevation and malaria such that the biology of malaria vectors with species diversity and density declines from the lowlands to highlands.^66^ Increasing distance from a village center and decreasing elevation were positively associated with malaria risk.^67^ In particular, the study in Yingjiang County in the Yunnan border area reported that the two main malaria vectors, *Anopheles minimus* and *Anopheles sinensis*, spread across different elevation levels and remained stable during the entire epidemic season in low-elevation areas along the border. The seasonal abundances of the two main vectors were different; whereas *An. minimus* preferred low-elevation and tropical areas with high density in July, *An. sinensis* preferred medium elevation with high density in August.^68^ The results of that study showed that the community structure of *Anopheles* was highly complex in areas below an elevation of 600 m, whereas a high elevation (> 1,800 m) correlated with low species richness, diversity, and evenness in the area.^68^ Based on studies of malaria vectors and their ecology in Yunnan Province,^69,70^ areas in this study at low elevation above 600 m were considered as having high risk for malaria occurrence; the risk decreases with the elevation increase up to 3,500 m where the *An*. *sinensis* could still be found in some areas, and areas over 3,500 m were classified as having the lowest risk of malaria transmission.

Related to elevation is the slope factor; steeper slopes allow the fast movement of water and, thus, result in less chance to accumulate stagnant water.^28^ Water body is a predominant risk factor for malaria transmission because it can form vector-breeding sites.^50,71^ Previous studies reported a positive association between malaria prevalence and proximity to water body, together with increased mosquito density and malaria.^72,73^ Larval mosquitoes are usually highly aggregated in pools of waters with specific characteristics.^28^ A study in Africa reported a significant risk factor for malaria risk was distance from household to stagnant water body, and households using a borehole/unprotected well in their yards.^74^ A study of malaria hazard mapping with climatic and geographic factors reported that almost all breeding places were located in riverbeds and riverbanks.^49^ In a mathematical model study, location far from water bodies was associated with malaria transmission seasonality, closely following rainfall, with a lag of 1–2 months.^75^ A study carried out in China indicated that populations living within 60 m of water bodies had a higher risk of contracting malaria.^12^ Based on studies indicating that mosquitoes fly no more than 170 m after ingesting a blood meal^76^ and that a hungry mosquito will fly up to 1,500 m,^77^ in this study areas above 1,500 m from water bodies were classified as low-risk areas.

The integrated effect of land cover and land use as anthropogenic elements, as identified by the experts in this study, was forest coverage, cropland, and urbanization. Previous studies in the Greater Mekong Subregion suggested that forest was commonly considered as a major determinant of malaria risk; a large proportion of all malaria cases and deaths occurred in the central mountainous and forested areas in Vietnam,^78^ and about 60% of the total malaria cases occurred in forest areas in Myanmar.^79^ Studies along the Thailand–Myanmar border also noted that the seasonal abundance of mosquito vectors and the seasonal movement of local people to forests, either to find forest products or to carry out agricultural activities, with overnight stays on either side of the border, can increase the risk of contracting malaria.^80,81^ A study in India using both a model and observations also reported that malaria vector abundance is higher during the summer monsoon season, and the intensity of malaria transmission is higher with the mosquito populations and the number of infective bites, particularly in forest or mountainous ecotypes.^82^ Contrarily, other studies reported forest clearance which provides abundant new habitat for vector species is a classic cause of the emergence of malaria problems,^83^ and vectors are found within 5 km of deforested areas.^26^ A study in the Amazon area also suggested that deforestation and other human environmental alterations had changed in the presence of both mosquito larvae and adults; although forest clearance and pollution may reduce the availability of larval sites for one species, it may conversely increase habitats preferred by another species.^50,84–86^ A study in the forested area of northern Myanmar bordering with China suggested that the scenario was different from other parts of Southeast Asia such that the high altitude leads to low temperature and thus less malaria vector, whereas malaria vector normally occurs in lowlands, foothills, and mid-hills some distance away of forest.^87,88^ According to the experts in this study, forest was not suitable for mosquito proliferation. Unfortunately, with some limitation of data availability in this study, the developed model in this study was based on the forest factor coded as a binomial variable (0 or 1) rather than the distance to forest. Further spatial studies of changes to forest areas over longer temporal data periods may result in an even better best-fit model.

Deforestation and cultivation of commercial crops have created major environmental changes in rural areas of Southeast Asia and China.^89–91^ After 2005, in Yingjiang County, Yunnan Province, the main crop changed from rice to bananas, and in early 2016, the area became a source of banana production, and no rice crops remained.^68^ A few vector studies reported that such changes suggested alterations in the population density, life history, and behavior of vectors.^68,71,92^ In the study areas, the subsistence activities of local residents and several ethnic minorities were associated with forest logging and rice, or banana and rubber planting. According to the experts, the cropland was considered as a risk factor that affects malaria incidence because rice, banana, and other irrigated crops in the study areas often create an ideal habitat for the mass production of mosquitoes. Previous studies in the same study areas reported that the peak times of malaria occurrence were closely related to agricultural activities; farmers cultivate crops in summer and harvest in autumn, when mosquitoes propagate actively with feasible climate and ecologic environment, while the farmers usually worked in the field and slept in the open.^56,57^ In addition, the cropland in the study areas could also be changed because of several dam development during the study period. As noted in the literature, the construction of dams for irrigation or hydroelectric power can lead to high populations of *Anopheles*.^38,93^ In a study in Africa, an increase in malaria incidence in villages around dams at all elevations was found to be significantly associated with reservoir-associated factors (distance from reservoir shoreline, monthly average reservoir water level, and monthly water level change), and reservoir water level management could help manage malaria transmission around dams.^94^ However, there were inconclusive results about cropland or agriculture areas, as shown in other studies on land coverage/use, where a positive association between agricultural activities and malaria risk was found for slash-and-burn agriculture but not in areas deforested for industrial agriculture.^50,54,55,91,92^ Again, due to limitations of data availability (the same for the other two factors, water body and forest coverage), the model developed in this study was based on cropland being coded as a binomial variable (0 or 1). The effect of cropland on malaria incidence thus needs further investigation; a more precise model might be obtained by incorporating longer periods of spatial and temporal data into the model.

Urbanization is another integrated land use and land cover indicator that is related to breeding sites and mosquito survival rate and dispersal.^28^ In rapidly expanding urban areas, extensive water storage and inadequate water disposal can lead to high mosquito populations. The absence of cattle can promote stable transmission by forcing zoophilic species to feed on people. Vectors are absent in urban areas but found in the urban periphery where the settlements retain rural characteristics, and the dense populations promote conditions that are ideal for transmission.^26,38^ Many studies of the impact of human population and urbanization on *Plasmodium* malaria endemicity reported a general trend of reduced transmission in urban areas in Asian and African countries.^95–98^ A study in Cameroon evaluating a mathematical model of malaria transmission noted that besides climatic factors, transmission tends to be high in rural and peri-urban areas relative to urban centers; however, one should be cautious that the model might be oversensitive by neglecting population movements and differences in hydrological conditions, housing quality, and access to health care.^75^ In the model developed in this study, the assumption was set for the lowest risk of malaria infection when the distance away from central urban area was within 5,000 m.

Related to urbanization, population density was found to be an important predictor of malaria risk as it provides a reliable metric to adjust for the patterns of malaria risk in densely populated urban areas.^99^ A study in the Peruvian Amazon, using boosted regression tree models based on social and environmental predictors derived from satellite imagery and data, noted that cumulative rainfall, population density, and time to populated villages were consistently the top three predictors for the incidence of both *Plasmodium vivax* and *Plasmodium falciparum*.^100^ Population density was thus considered as risk by the experts in this study under the assumption that the denser the population, the more vulnerable it will be to malaria hazard; when population densities are high, there is a greater likelihood that malaria will be transmitted.^27,28^ In this study, the density unit was people per hectare; the minimum and maximum values were 0.005 and 104.539 unit, respectively. It should be noted that socioeconomic factors were not directly put into the model according to the experts in this study. Rather than considering socioeconomic status, which is a factor at the micro- or individual level, the experts selected to consider risk factors at the macro level which could be observed via population density and other related risk factors including population movement (introducing imported cases), health facility distribution, and urbanization.

Disease risk depends on the spatial connectivity of habitats for vectors and hosts; geographical and environmental features generally control the dispersion of vectors from their breeding sites to hosts.^51^ However, access to health care as a specific factor related to host alone could be an incremental risk factor for disease transmission. Access to health care can be defined by different dimensions of access, including availability, accessibility, affordability, and acceptability; some have defined access as the opportunity to use health care, whereas others did not distinguish between access and use.^101^ Several studies on applying environmental variables that influence the transmission of malaria had applied and reported best-fit models with distance to streams, distance to main road, distance to health facility, and distance to border.^32,102–104^ The experts in this study identified two proxy indicators of access to treatment and care, distance to road and distance to health facilities. Distance of a place from roads could affect or determine the effectiveness of measures to be taken to control the risk of malaria infestation.^27^ A study in Kenya found that malaria hotspot locations correlated with environmental and static household characteristics, such as distance to roads or rivers.^105^ In the border areas of China, poor transportation still exists, making it difficult to perform blood smear verification within 3 days.^63,106^ A study in Henan Province, China, using surveillance data during 2012–2017, reported that imported malaria cases were mostly working-age males, and many typically went to provincial and municipal healthcare institutions as their first option because many were near cities on their way home; however, cases living away from cities, in rural areas, tended to frequent local healthcare institutions nearby.^107^ As patients in rural areas usually have a long way to go to county healthcare institutions, they usually take empirical medicines instead of visiting distant healthcare facilities.^107–109^ In this study, an assumption was that the shorter the distance from the patient’s house to road, the higher the probability of access to prevention and control; thus, the malaria risk on a community is lower. A previous study in Zimbabwe assumed lowest risk within 5,000 m,^110^ whereas in Mozambique, the lowest risk was classified within 2,500 m.^32^ This study adopted the assumption of the lowest risk of malaria infections when the distance from road access was within 5,000 m.

Distance from the patient’s house to the nearest health facility was another risk factor for malaria as it reflects the quick and effective provision of malaria control and treatment.^28,111^ A systematic review study noted that 77% of the included studies showed evidence of an association between worse health outcomes the further a patient lived from healthcare facilities.^112^ A study in Nigeria reported poor access to healthcare and public health services could be responsible for the high malaria endemicity in the region.^113^ Besides access to health facilities, a study in Uganda noted that facility quality and readiness were important factors associated with reduced risk of malaria outcomes.^58^ A few studies reported that the degree of severity of malaria in patients was due to disparities in the availability of health services and significant gaps in the awareness of malaria at different administrative levels.^107,114,115^ Although China has made remarkable progress in strengthening its primary healthcare system, challenges still exist in the structural characteristics and quality of care, which affect the efficiency of care delivery.^116^ Based on the model developed from the surveillance data in Henan Province, China, major factors influencing complications of imported malaria cases were due to inadequate seeking of medical care and insufficient capacity to diagnose malaria by healthcare institutions at lower administrative levels.^107^ The results of that study noted the time gap between onset and initial diagnosis among 77% was no more than 72 hours; however, those visiting county healthcare facilities had the longest period from onset to initial diagnosis, compared with those visiting private clinics and township health facilities.^107^ Similarly, a study comparing the same time gap among imported malaria cases in former endemic and non–malaria-endemic areas in China reported that health facilities in former endemic areas outperformed those in former non-endemic areas.^60^ Other studies in China categorizing the foci and evaluating whether the response met the requirements issued by the country during the same period of this study reported that the healthcare workers’ response in these foci was inefficient.^106,117^ Regarding distance to health facility, a study in Africa reported that more severely ill cases traveled further for clinic visits, and the rate of clinic visits decreased linearly with the number of kilometers from the residence to a clinic, even after adjusting for some potential confounders (such as socioeconomic status, maternal education, distance to a road, and household clustering) in the analysis.^59^ Another study in Africa reported residing beyond 10 km from the clinic to be associated with higher prevalence of comorbidities and the incidences of severe malaria and severe illness among children with acute malaria.^118^ In Yunnan, township health facility is the lowest level unit in which the malaria cases are diagnosed, treated, and reported simultaneously. There is typically one health facility per township in the study area. Spatially, the location of these health facilities was indicated on a map, and the average distance from the nearest health facility was within 20,000 m.

The challenge for primary health care in China requires a strategy aiming for equity in access. In 2014, the Chinese government issued official guidance emphasizing that primary healthcare institutions are responsible for providing basic health services to migrants within China.^116^ The health facilities at all levels in the study area provide treatment for malaria patients who are local residents or migrant workers. Technical Scheme of China Malaria Elimination classified two types of malaria cases: “indigenous case” and “imported case.” An imported case is a diagnosed malaria patient with a history of traveling to overseas malaria-endemic areas during the malaria transmission season, and the onset of malaria symptoms was less than 1 month after return. An indigenous case is a malaria patient with infection acquired from local transmission within China or a malaria case with no clear evidence of being imported from outside the country. Importing infections must be addressed to achieve malaria elimination.^119^ From an elimination setting perspective, an opportunity to prevent acquisition or transmission of imported parasites can be tackled during four general stages of human movement: while people are in the eliminating region, during transit, in the endemic region, and on return to the eliminating country.^119^ The magnitude of imported malaria infection in terms of timing and number is a function of several factors, including the transmission intensity of the origin location, the number of people going to and from that location, the activities undertaken in the location, and prophylaxis availability and adherence.^61,120^ Several studies have indicated that Chinese laborers returning to China have mainly contributed to the increasing importation of malaria into China.^61,121–123^ Regarding border malaria, as there is generally no natural barrier along the border lines, it is open to a large mobile cross-border population, making management of imported malaria a significant challenge.^63,124,125^ A study in Yunnan Province noted the differences in seasonal pattern of imported cases in different locations, which may be due to the different cross-border behaviors of people.^2^ Other studies in the same areas of this study also indicated imported malaria cases have been a major challenge of interrupting malaria reintroduction in China.^1,62,117,126^ A study on receptivity to malaria at the Yunnan border noted that under conditions of high receptivity and potential exposure of the local people, imported malaria cases in the county could increase the probability of reestablishment.^68^ The existence of disease ecology and mosquito species helps malaria transmission from imported cases to local people.^127^ With similar climate and natural environment and vectors, importation and autochthonous transmission of malaria have been found in the regions along the China–Myanmar border.^7,61,124^ The endemicity of malaria was found to be positively related to the distance between the households of the cases and the nearest larval habitats.^12,128^ As high importation of malaria and wide distribution and abundance of malaria vectors in the China–Myanmar border area sustain risks for secondary infections among local populations, in this study, risk factor as distance to imported cases was defined as the mosquito flight range and the sphere of malaria patient activity. As noted in the literature, the flying distance of different types of mosquitoes can be kilometers, under advantageous geographical, wind velocity, and other meteorological conditions.^129,130^ The assumption was set such that the highest risk occurs within 5,000 m between imported cases and indigenous cases in a spatial pattern.

Based on the risk map developed by the MCDA model, spatial heterogeneities still remain presented over the years in the border areas of Yunnan Province. Counties with high malaria risk are mainly concentrated in the central part (west of Yunnan) that borders with Myanmar. Besides the declining trends, the fast declining rates of indigenous (99% reduction; from 145 in 2011 to one in 2016) and the relatively slower decreasing trends of imported cases (58% reduction; from 432 in 2011 to 183 in 2016), most of the reported malaria, particularly the imported, cases persistently clustered in certain locations, as shown in the developed spatial risk maps. According to the literature and the highest factor weight rated by the experts in this study, the imported cases were identified as a major factor contributing to persistent malaria transmission in the border areas of Yunnan Province. As shown in this study, when taking into consideration the imported cases as a time-varying factor, spatiotemporal MCDA risk maps were developed annually. Despite deceasing reported malaria cases, the MCDA risk maps of annual imported cases combined with other eight risk factors showed somewhat consistent risk locations within the 18 counties over the study period of 2011–2016. It is important to focus the elimination efforts in the specifically identified locations with high risks, as shown in the fine MCDA risk map.

### Limitations of the study.

There were several limitations in this study. First, the use of the spatial MCDA method might be useful for exploration of factors related to complex epidemiology in making decisions on malaria management and control strategies, especially in moving toward elimination phases, but there are several approaches in creating MCDA models, and the model used in this study might be effective or appropriate with a certain setting, not for all situations. It was suggested in the literature that MCDA-based models might have the potential bias for manipulation of the decision result; thus, one should take into consideration the sensitivity and robustness of results and should use the results of the model as a decision aid support rather than a decision-making tool.^4,19^ Second, it should be noted that the MCDA models are basically not designed and cannot be used to conclude about the causality factor. Third, in identifying risk factors, the MCDA model used in this study was based on navigating and synthesizing the input provided among the experts during the consensus process, and, thus, they could be subjective and sensitive to inaccuracies or omissions of important factors. Moreover, a bias could be related to the subjective nature of the MCDA approach in assigning fuzzy functions and weights to the selected factors.^23^ Fourth, in creating a risk map in this study, the numbers of positive malaria cases per village were located at the center of the village, not at the specific coordinates of the location of each positive malaria case where the actual infection might have occurred. Correlation between imported and indigenous cases in this study was based on spatial pattern over the years, not truly matching space and time of each individual case. A bias could also occur for the factors related to land cover/use (forest, cropland, and water body) in this study as they were assigned with value 1 or 0 (exist or does not exist) in the spatial coordinates, not distance to them. Finally, there was a suggestion in the literature that MCDA requires the participation and engagement of a number of experts; thus, it is more suitable for long-term planning rather than during an emergency/outbreak situation. This study, however, created the spatial MCDA model to assess the effects of major risk factors and aid the malaria planning for the next few years in an attempt toward malaria elimination in Yunnan border areas.

## CONCLUSION

During the pre-elimination period, several strategies and interventions have been implemented. However, malaria transmission along and across borders remains a great threat to the successful elimination of malaria in China. To move forward and eliminate malaria as planned, the main focus should be appropriate control in areas particularly affected by imported malaria. The study has demonstrated the novel integrated use of spatial MCDA to model the malaria epidemic at the village level. The model could not only serve as a predictive tool for identifying and analyzing malaria risk factors but also be used to obtain a comprehensive understanding of the dynamics surrounding malaria in the construction of surveillance–response systems. The MCDA model and risk maps developed in this study could enable a visualization of the effects of different proposed factors to the malaria situation in the study area. The incorporation of factors identified in this study together with dynamic changes in imported cases in border areas over the years has provided additional detail to the risk maps relative to past studies that did not use the MCDA model. The proposed model could help public health decision-makers/policy-makers to give additional attention to spatial planning for cost-effectiveness and prevention and control strategies gearing to the specific location in the risk areas shown in the fine risk maps.
